# Monthly “Esquisse” of Parisian Medicine and Surgery

**Published:** 1859-04

**Authors:** Theophalus Mack

**Affiliations:** Lecturer on Materia Medica and Therapeutics, in the Medical Department of the University of Buffalo


ART. II.—*Monthly "Esquisse" of Parisian Medicine and Surgery.* By THEOPHALUS MACK, M. D., Lecturer on Materia Medica and Therapeutics, in the Medical Department of the University of Buffalo.

[Under the above caption, I propose, Mr. Editor, to send you a monthly sketch of the most interesting items in the French journals of the present year, as they shall appear. I shall commence at once with January, having just now received all my periodicals for that month. In this way, I hope to be able to present to your readers, promptly, such discoveries, opinions, and general advancement in the science termed medical in that country, as it is incumbent upon every practitioner to be thoroughly conversant with.]

A very interesting discussion has been sustained at the "Academie des Sciences," between two of its most distinguished members, MM. Desprets and Dumas, upon "The Composition of Bodies, hitherto Reputed Simple." We merely allude to the fact of this question being brought up very forcibly, no practical benefit having so far ensued.

The question of spontaneous generation was discussed at the *séance* of the third of January. The *savants* were unanimous in condemning heterogenesis.

M. Castorani labored to show that the various affections of the cornea, known under the general denomination of keratitis, are produced by the penetration into the cornea of abnormal secretions from the conjunctiva.

M. Claude Bernard maintained that the placenta is destined, in the first period of the fœtal development, to accomplish the glycogenetic function of the liver.

M. Petrequin announced the cure of a large hydrocele of long standing, by galvanism, with a single pair of Bunsen's battery. The indication, he says, being to stimulate the vitality of the tunica vaginalis, and reëstablish the equilibrium between absorption and secretion, he obtained it for his patient, by applying one pole of the battery upon the base, the other upon the summit of the hydrocele. The sitting only lasted half an hour. He was then put to bed, and the next day his hydrocele had disappeared. A moderate compression was kept up, internal treatment, also, and now, after a month, he can demonstrate that it is a radical cure.

M. Bozeman, one of the principal promoters of operations for vesico-vaginal fistula, has converted M. Robert at the Hotel Dieu, who was previously sceptical, from the fact, that notwithstanding the important labors of Jobert, the success in France of such operations had been the exception. The honor of the initiative in successful operations is very properly given to America; the English operators having wisely adopted the American method, and, as in the hands of Mr. Baker Brown, of London, with the happiest results. One paragraph, however, of Mr. Robert's article I must transcribe; it is too good to be lost, and shows how utterly "at sea" our trans-Atlantic *confrères* constantly prove themselves to be, respecting American practice:

"We should say, at first, that vesico-vaginal fistulæ, rare enough in France, are, on the contrary, frequent in England and in America (?) on account of the position, which, in those two countries, women are made to take during parturition. They make them *sit upon an arm-chair (fauteuil)*; the pelvis is then in a dependent position, and the head of the fœtus presses strongly upon the vaginal walls; hence, *fistulæ are frequent.* Surgeons, therefore, have numerous operations to make, and it appears that Mr. Baker Brown, of London, occupies himself specially with this part of surgery." (*Gaz. des Hôpitaux.*)

The patient reported appears to have been a very unpromising case for the operation, the fistula being very large, and the operations by simple suture, and by the autoplastic process of Gerdy, having been successively tried, and having failed, M. Bozeman proceeded to unite the edges with ten points of suture with silver wire, without any lateral incisions, the edges only being freshened. The advantages of the American operation are: 1st. The silver sutures; 2d. The position of the patient, on the knees flexed upon the pelvis, and the elbows, the head low; 3d. The speculum being in the form of a curved gutter, made of copper, plated; 4th. The greater extent of surface made raw, even extending to sound portions of the vaginal mucous lining; 5th. The curved scissors; 6th. Not permitting the suture to pass through the mucous membrane of the bladder; 7th. The short interval between each suture; 8th. The plate of lead, through holes in which the sutures are finally secured, compress the wound uniformly; it avoids the contact of liquids with the wound, and it supports the rings of lead which replace knots, and thus prevents irritation; lastly, the cares consecutive to the operation have a much greater importance attached to them in the American process. One thing more, the *decubitus dorsalis* is recommended to be strictly maintained, and the sutures to be allowed to remain ten days.

A case of hallucination in a child, ætat five years, convalescing from pleuro-pneumonia, is reported, with the following remark:

"I have already reported numerous examples of hallucinations observed during the course, and, above all, at the decline of acute pneumonia. In some instances we could attribute this complication to the abuse of alcoholic drinks. Here this cause could not be adduced. It must be admitted that the acute disease was sufficient to produce it. As we have seen, the hallucinations manifested themselves, when all the symptoms of the pneumonia had disappeared, and in the midst of a complete apyrexia. They appeared to have been produced by debilitating causes: privation of nourishment, loss of blood, &c. They ceased finally upon the administration of some spoonfuls of an opiate potion, and light aliments."

A case of hysteric trembling, simulating chorea, was admitted to the Hotel Dieu, Dec. 7, ult.; the diagnosis established by the general hysteric group of symptoms. The patient was immediately submitted to prolonged baths; every day she passed two or three hours in a large bath of tepid water, and in fifteen days the cure was complete.

A "triple operation for cataract" has been performed by Dr. Magne, with cure. Both eyes were couched successfully, but about fourteen months after the operation, the cataract was thrown into the anterior chamber in one, where it remained in front of the iris, masking the pupil, until it was dexterously extracted with a curette, piecemeal through a puncture of three millimètres at the external part of the cornea. Ice is highly lauded for its assistance in this case.

M. Bosel obtained a cure of salivary fistula, by obliterating the duct of Steno.

A rare example of general tuberculosis, at the *Hôspital Beaujou*, occurred in the service of M. Barth. The diagnosis was pulmonary tuberculisation, with degeneration of the abdominal and pelvis ganglia; tumefaction of the knee developed under the same influence. Upon autopsy, the pia mater was covered with tubercles, about the size of grains of coarse wheat flour, dry adherent pleuræ; lungs infiltrated with tuberculous granules, pericardium glued to pleuræ; the walls of the heart are hypertrophied, and adherent, by a fibrous reddish substance, strewn with tubercular granulations; tubercles in the mesenteric glands, peritoneum, glands of Peyer, lumbar, and iliac glands, liver and kidneys. The knee presented tubercular deposit in the fatty tissues which unite the crucial ligaments. The serous surface of the capsule of the joint was studded with grains of the same nature.

At the *Maison de Santé*, an adenoid tumor was removed from the female breast, having returned five times in the course of six years, non-malignant, and having caused no trouble to the general health.

A case of blue coloring of the skin has been observed near Colmar, on the Upper Rhine, in the person of a blonde, aged nineteen; *chromhydrosis*, probably consisting in a "secretion of animal indigo, by the sudoriparous glands."

M. Robert has drawn attention to the accidents resulting from the development of the wisdom teeth; ostitis, and periostitis of the maxilla, neurosis, inflammation of the adjacent soft parts, migratory abscesses in the throat and neck, ulcerations of the gums, and abscesses, below the chin, &c.

A case of death, consequent upon the inhalation of chloroform, occurred at the *Hôspital Saint Louis*, on January 15th. It was administered to aid in the reduction of a dislocated shoulder. The quantity poured upon the compress for inhalation did not exceed fifteen to twenty grammes. The radial pulse, up to the moment of its cessation, did not furnish any particular indication. The reduction was effected in about three minutes, and in about five the patient was dead. Post-mortem examination only showed a peculiar flaccidity of the heart, and a want of consistence in the muscular fibres, which could be torn by the least pressure of the finger.

Legrand du Saulle describes a very important sign in the medico-legal history of the attempt upon chastity of little girls—a crime we find increasing lately. This sign is the infundibuliform depression of the vaginal orifice, observed twenty-nine times before the age of eleven years, twenty six times between eleven and fifteen, four times between fifteen and twenty, and once in a woman of forty-one. M. Tardieu has verified this sign in sixty cases out of one hundred and eighty-one criminal attempts upon women; they are given above. In the case at forty-one, there was an abnormal contraction and rigidity of the vagina.

At the Hospital of Tenes, Algeria, M. Serni reports a case of albuminuria, complicated with incomplete amaurosis and eclampsia. At the Sectio Cadaveris, in addition to other usual pathological appearances under such circumstances, an elongated lamella of bone was situated between the folds of the falx cerebri. He regards it, however, as a coincidence, attributing all the symptoms to the disease of the kidneys.

M. Rocque, in an inaugural thesis upon the "*menopause*," (the climacteric period), establishes a similarity between the rise and decline of genital life. He carefully distinguishes between diseases attributable to the *menopause*, and those incident to the age of forty or fifty years.

M. le Docteur de Lamare has taken the possibility of the contagion of phthisis into consideration, and arrives at the following practical conclusion: that without declaring phthisis to be contagious, it is better not to multiply points of contact with phthisical patients, such as occupying the same bed, &c.

In the *Académie de Médicine*, M. Malgaigne has had a very hot engagement with M. Trousseau, as to the value of tracheotomy in croup, and the substitution of "*tubage of the larynx*." The *quæstio vexata* of tracheotomy brought forward a discussion replete with practical instruction upon the operation, and the cares consecutive, necessary to ensure favorable results. Some bold expressions of doubt, as to the cypher of successful cases reported, having been collated only from cases where the operation was imperative, other treatment having failed. The conclusions at the close of this debate were: 1st. The tubage of the larynx, as it has been applied up to the present time, has not appeared to us sufficiently useful, or enough exempt from dangers, to merit the *approbation* of the Academy. 2d. Tracheotomy, in the actual state of the science, is the only mode of procedure, when there remain no other chances of safety in the employment of medical measures.

Chirurgical Society. Illustrative of the reproduction of bone by the periosteum, M. Larrey read two observations; one of necrosis of six inches of the femur, (shaft) enclosed in bone of new formation, which, from the report of the case, appears to have been secreted by the periosteum. Two applications of the trephine, and the use of the gouge, enabled the operator to extract, in fragments, a sequestrum of the length mentioned. A second observation was that of a ball penetrating to the medullary canal of the tibia at its upper end, and after remaining four months, being removed by the trepan, at the lower end of the bone, about two inches above the malleolus internus. Here the ball had destroyed the internal wall of the tibia, the vessels and nerves of the medulla, in fact, all the elements of nutrition of the tibia. Hence vitality could not have been conserved to this portion of the bone, except through the means of the periosteum.

The laryngo-tracheal apparatus of a patient who had worn a canula in the larynx for about one year, presented the following appearances:

1st. The fistulous opening giving passage to the canula, the skin becoming continuous with the mucous membrane by a transition analogous to that which takes place at the opening of the nares, smooth and clean.

2d. This opening, seated in the crico-thyroidean membrane, which has preserved its characters. (The operation was performed in consequence of the transfixion of the larynx from right to left by an iron fork.)

3d. The thyroid cartilage formed an angle more acute than in the normal state; its two quadrilateral portions are movable upon each other, and united by a fibrous tissue along the vertical prominence of its anterior face. The mean notch, in its inferior border, is much greater than usual, and has increased at the expense of the right quadrilateral portion.

4th. The arc of the anterior circle of the cricoid has much increased in volume. The external face is rugose, as if crusted with newly formed cartilage, and presents, on the left side, a little cartilaginous tubercle, very prominent.

5th. The cricoid and thyroid cartilages are immovable one upon the other. This man had never been able to pass more than three or four hours without his canula, from the difficulty of respiration.

A case of *complete* and *simple* luxation of the carpus upon the forearm was reported.

M. Gosselin cited a recent case of aneurism, or pulsatile tumor, of the left tibia, cured by ligature of the femoral, compression having failed.

At the Society of Practical Medicine, the principle that no age should be fixed for weaning infants, but the progress of dentition should alone be our guide, was maintained.

A frightful and inexplicable accident befel a physician, M. Causé, residing at Budesheim. Desiring to light a cigar, a little piece of burning phosphorus fell upon his hand, and caused a burn over one of the phalanges. The suffering became so severe, that the doctor incised the part himself freely, and created slight hæmorrhage. This measure being ineffectual, he had his finger amputated. This operation did not produce the result hoped for, and in a consultation of several physicians, amputation of the arm was judged indispensable. The unfortunate doctor, after courageously submitting to this new operation, died in a few hours. Although the poisonous property of phosphorus is well known, this sad catastrophe should serve as a terrible lesson.

Of the recent steps in advance made by science, cheapening the elements in the Voltaic circle must prove of great practical utility; thus a cylinder of lead is made to replace the zinc in the Bunsen battery, and the salt of lead formed has a commercial value. MM. Fonvielle and Humbert form a constant and economical battery after the following manner: They place jars, containing each a galvanic pair of zinc and charcoal, in a vase, hermetically sealed. The vases are furnished with two orifices; one inferior, through which chlorine is admitted, and one superior, for its exit, as it is generated from the liquid. The gas traverses the exciting liquid in the closed piles, and maintains it in a constant state of concentrated chlorination.

Marsh's apparatus is insufficient for the detection of small quantities of antimony; it is necessary to resort to incineration to discover minute traces of this metal. The filtration of air through a layer of pulverized charcoal has already been applied successfully. The charcoal does not act as an antiseptic, strictly speaking; it absorbs the emanations, submits them to a slow combustion with the oxygen its pores are filled with, which changes the carbon into carbonic acid, and the hydrogen into water.

Cutaneous anæsthesia is a frequent symptom, according to M. Voisin, of hysteria, most frequently after a free attack of that malady, a diminution, and often absence of cutaneous sensibility. If the immediate cause of hysteria has its seat in the brain, and this paralysis of the cutaneous nerves results from the loss of consciousness, there would appear to exist a relation of cause and effect between loss of consciousness and anæsthesia.

At the *Charité*, a young girl, aged 17, shows an enormous hypertrophy of the mammæ, thus described:

"The breasts of this young girl represent two enormous pedunculated appendages, falling over, and covering the chest and belly, down to the pubis. Measured in their greater diameter, they are seventy-five centimètres for the left, and seventy-two centimètres for the right. The circumference of the pedicles was about fifty centimètres. Their weight, as far as it was possible to appreciate it, was about six and a half kilogrammes for the left, which appeared a little more developed. The skin covering these enormous mammary glands, (for, as we shall see at the right time, we had not to deal with either cancers, or adenoid, or lipomatous tumors, but just with a simple hypertrophy of the glandular tissue, as well as of all the anase, cellular, skin, &c., which entered into the abnormal constitution of the breast); the skin, we say, did not appear, at any point, to have undergone any alteration, or any modification in its texture; it was white, soft to the touch, elastic, mobile upon the subjacent parts; in a word, it offered all the characters of the normal integument of the breast; it had acquired a gradual development, proportional to that of the gland, and of the other parts of the breast; consequently, it is neither thickened and hypertrophied, as in elephantiasis, for example, nor chapped, or rendered thin, as the integuments generally are, which have suffered a considerable and rapid distension. It was not drawn *(tiraillée)*, save at the commencement of the breasts, at the pedicles, there where she supported all the weight of the breast in standing. The nipple did not exist, or, at least, it was flattened, and almost entirely effaced, but its place was perfectly indicated by the areola of a brownish tint, and extremely large."

Menstruation had been suppressed for six months. Amputation was performed upon each at an interval of twenty-one days. The hæmorrhage from the very much enlarged veins, gave some trouble, but the operations proved successful; the menses reappeared, and she departed on her way rejoicing.

Dr. Legouest is energetic in the laudation of injections of chloride of zinc, 1 part in 1000 in solution, in the treatment of urethritis. In diptheritic angina, a correspondent recommends immediate ablation of the tonsils, if at all hypertrophied, or indurated. To abort the disease during its pharyngeal period by ablation or cauterization, is certainly the most rational therapeutic method.

At the Chirurgical Society, M. Desomaux sustained the propriety of operating for hare-lip upon newly-born infants, and advanced two successful cases to sustain his position.

M. Becquerel denies the fungosity and granulations of Recamier in the inner surface of the body of the uterus, and attributes all the improvement consequent upon the use of the curette *(grattage)* to a certain degree of acute inflammation, superinduced, by the use of the instrument, upon the existing chronic inflammation of the mucous membrane.

M. Follius' presentation of a patient, affected with contraction of the anus, in consequence of the operation for hæmorrhoids by the ecraseur, gave rise to a discussion in the Chirurgical Society, as to the use of that instrument, and the mode of operating. The utility of the instrument was fully sustained, but the lateral or partial section of the tumors was maintained to be preferable to the circular method.

M. Huguier demonstrated that enterotomy for imperforate anus should be practised in the right inguinal region, and not in the left, the operation of Littre. The illiac flexure of the colon in the fœtus and newly-born infant is found to the right, and not to the left. This fact is also of consequence in the reëstablishment of the anus in the normal site. In place of searching for the rectum behind, and to the left, as generally practised, our researches should be directed *in front, and to the right.*

At the *Académie des Sciences*, M. Halma-Grand read a memorandum, having for title "The Conditions, Physical and Chemical," which should preside in the composition of every febrifuge, as a substitute for sulphate of quinine, and especially of the ferrocyanide of sodium, and of salicine. The properties are bitterness, the ordinary attribute of tonics, and an azotized composition, which ensures the facile absorption of the medicament.

